# Prognostic value of cerebral venous outflow profiles for outcomes prediction following reperfusion therapy in acute ischemic stroke: a meta-analysis

**DOI:** 10.1007/s00234-025-03688-w

**Published:** 2025-07-08

**Authors:** Hesham Kelani, Mohamed R. Abdelraouf, Shree Rath, Shrouk F. Mohamed, Hazem Mohamed Salamah, Qasim Mehmood, Muhammad Ansab, Danisha Kumar, Ahmed Abd Elazim, Diana Greene-Chandos, Ketevan Berekashvili, Ambooj Tiwari, Volodymyr Vulkanov, David P. Lerner, Eytan Raz

**Affiliations:** 1 Neurology Department, SUNY Downstate Health Sciences University at One Brooklyn Health, Brooklyn, NY USA; 2 Faculty of Medicine, Alexandria University, Alexandria, Egypt; 3 All India Institute of Medical Sciences, Bhubaneswar, India; 4 Faculty of Medicine, Zagazig University, Zagazig, Egypt; 5 King Edward Medical University, Lahore, Pakistan; 6 Services Institute of Medical Sciences, Lahore, Pakistan; 7 Dow University of Health Sciences, Karachi, Pakistan; 8 Department of Neurology, Sanford USD Medical center, Sioux Falls, SD USA; 9 Department of Neurology, School of Medicine, University of Saint Louis, Saint Louis, MO USA; 10 Department of Neurology, Mohawk Valley Health System, Utica, NY USA; 11 Chief of Neurology, South Brooklyn Health, Clinical Assistant Professor YNU Grossman School of Medicine, New York, NY USA; 12 Department of Neurology, Rutgers New Jersey School of Medicine, Newark, NJ USA; 13 Department of Neurosurgery, NYU Langone, New York, NY USA

**Keywords:** Venous outflow, COVES, Biomarker, Reperfusion therapy, Intracranial hemorrhage, Thrombectomy, Acute ischemic stroke

## Abstract

**Background:**

Recent studies have suggested that favorable venous outflow (VO) may be a promising imaging biomarker to predict clinical outcomes following reperfusion therapy in patients with acute ischemic stroke caused by large vessel occlusion (AIS-LVO).

**Methods:**

A comprehensive literature search was conducted in PubMed, Scopus, WOS, and Cochrane to identify studies that evaluated VO profiles, assessed using the Cortical Vein Opacification Score (COVES). The risk ratio (RR) and 95% confidence interval (CI) for the outcomes, including functional independence, assessed by modified rankin scale at 90 days (mRS 0–2), hemorrhagic infarction, parenchymal hematoma, 90-day mortality, intracranial hemorrhage (ICH), and symptomatic ICH (sICH), were calculated and analyzed using the ‘meta’ package in R version 4.4.1.

**Results:**

A total of six studies encompassing 2249 patients were included. Patients with favorable VO had a higher likelihood of achieving functional independence at 90 days (RR = 2.15; 95% CI: 1.35, 3.42; *p* = 0.01) and a lower incidence of 90-day mortality (RR = 0.39; 95% CI: 0.30, 0.51; *p* < 0.01), parenchymal hematoma (RR = 0.36; 95% CI: 0.27, 0.47; *p* < 0.01). Furthermore, sICH was less frequent in patients with favorable VO (RR = 0.39; 95% CI: 0.17, 0.89; *p* = 0.03). However, hemorrhagic infarction and any ICH did not differ significantly between the two groups (p-values: 0.06 and 0.32, respectively).

**Conclusion:**

VO is a promising imaging biomarker for predicting outcomes in patients with AIS-LVO following reperfusion therapy. Prospective clinical trials are warranted to investigate the predictive value of VO, assessed on multiphasic computed tomography angiography (CTA), as a prognostic marker in this patient population.

**Supplementary Information:**

The online version contains supplementary material available at 10.1007/s00234-025-03688-w.

## Introduction

In recent years, mechanical thrombectomy has emerged as a groundbreaking treatment for large vessel occlusion strokes, offering hope for improved outcomes in many patients. However, predicting patient outcomes following thrombectomy remains a critical challenge in clinical practice [1]. It is well established that good collateral circulation was associated with small infarct size, good functional outcomes, and lower rates of symptomatic intracranial hemorrhage (sICH) following thrombolysis [2].

Venous outflow patterns have garnered increasing attention as a potential predictor of thrombectomy success and subsequent patient recovery. This exploration into the role of venous outflow aims to shed light on its predictive value in thrombectomy outcomes for acute ischemic stroke patients. Understanding this relationship could have profound implications for refining patient selection criteria, optimizing treatment protocols, and ultimately improving clinical outcomes in managing acute ischemic stroke [3].

As previously reported [4–6], venous outflow (VO) was estimated by the cortical vein opacification score (COVES) on computed tomography angiography (CTA) images, which quantified venous opacification of the vein of Labbé, sphenoparietal sinus, and superficial middle cerebral vein on CTA as follows: from 0 to 2 points (0: not visible, 1: moderate opacification, 2: full opacification). Consequently, the sum of the scores of all three veins is quantified as COVES, ranging from 0 (no opacification of the three veins) to 6 (full opacification of the three veins). Recent studies by Adusumilli et al. [3], Faizy et al. [7], and Jiang et al. [8] have demonstrated that a favorable venous profile is linked to functional independence and excellent post-thrombectomy reperfusion in acute ischemic stroke patients with large vessel occlusion, resulting in lower incidence of intracranial hemorrhage (ICH) and good post-treatment outcomes. Furthermore, a favorable venous outflow profile has been shown to reduce the risk of developing cerebral edema, and ischemic lesion net water uptake (NWU), and have good functional outcomes regardless of arterial collateral status [7]. Moreover, unfavorable VO was associated with early neurological deterioration (END) and poor functional outcomes [9].

This systematic review and meta-analysis aims to comprehensively explore the relationship between venous outflow characteristics quantified by COVES on single-phase CTA and outcomes following reperfusion therapy in acute ischemic stroke patients. By synthesizing current evidence, we seek to elucidate whether venous outflow patterns can serve as a reliable predictor for functional outcomes, potentially informing clinical decision-making and improving patient selection for thrombectomy procedures.

## Methods

### Protocol documentation

This meta-analysis was conducted according to preferred reporting items of systematic reviews and meta-analysis (PRISMA) in adherence to the Cochrane Handbook for Systematic Reviews [10, 11]. The protocol for this study was registered on PROSPERO (International Prospective Register of Systematic Reviews; CRD42024609394).

### Data sources & search strategy

A literature search through PubMed, Cochrane, Scopus, and WOS was applied until October 23, 2024, without restriction to language or year of publication using the following terms (thrombectomy* OR embolectomy* OR “endovascular thrombectomy” OR “mechanical thrombectomy” OR endovascular OR “Vessel Reperfusion” OR thrombolysis) AND (stroke OR “acute ischemic stroke” OR “hemorrhagic stroke” OR “transient ischemic attack” OR “cerebrovascular accident” OR CVA OR “apoplexy” OR “cerebral accident” OR “cerebral infarction” OR “brain attack” OR “Cortical Vein Opacification Score” OR COVES) AND (“venous outflow” OR venous^*^ OR “poor venous outflow” OR “favorable venous outflow”). Supplementary Table S1.

### Eligibility criteria

Randomized clinical trials and observational studies that evaluated VO pattern determined by COVES in patients with AIS- -LVO who underwent reperfusion therapy without any limitations to language, publication time, gender, or age. However, studies that did not evaluate venous outflow, animal studies, and secondary research such as reviews and meta-analyses, letters, commentaries, editorials, and conference abstracts were excluded.

### Study selection

All citations were imported into Rayyan, a web-based software for de-duplication and initial screening. Two independent reviewers (Q.M., S.F.M) screened titles and abstracts for relevance based on predefined eligibility criteria. Eligible studies underwent full-text screening. Reference lists of included studies were manually searched for additional relevant articles. Disagreements at any stage of the screening process were resolved through consensus with a third reviewer (S.R).

### Data extraction

Two independent authors (M.A., S.F.M.) extracted the data from all included studies, following which all extracted data were reviewed by (S.R., M.R.A). The following data were extracted: year of publication, country, study period, study design, age, gender, baseline NIHSS, ASPECTS score, occlusion site, good arterial collaterals, and successful reperfusion “TICI 2b-3”. The authors independently extracted data for outcomes, including functional independence (Modified Rankin Scale at 90 days (mRS 0–2)), hemorrhagic infarction, parenchymal hematoma, 90-day mortality, intracranial hemorrhage and symptomatic intracranial hemorrhage (sICH).

### Risk of bias assessment

Two independent authors (D.K, S.R.) used the risk of bias assessment tools ROBINS-I and ROB 2.0 for non-randomized and randomized trials respectively [12, 13]. This tool can aid in identifying selection, performance, detection, attrition, and reporting biases. We categorized each domain’s contained articles as having low, some concerns, or high bias levels.

### Statistical analysis

Statistical analyses were conducted using the ‘meta’ package in R version 4.4.1. Dichotomous outcomes were pooled using the generic inverse variance method, with effect sizes expressed as risk ratios (RR). A random-effects model was employed to account for heterogeneity across studies. Heterogeneity was assessed using Cochrane’s Q statistic and quantified by the I² statistic. Statistical significance was set at a p-value threshold of < 0.05. A random-effects model with the inverse-variance (IV) method was employed to pool effect estimates. To identify the source of heterogeneity we conducted a leave-one-out analysis, which employed sequential exclusion of a single study from the meta-analysis and re-calculating the pooled effect estimate. To further explore potential sources of heterogeneity, a subgroup analysis was performed based on the classification of favorable VO as identified in each study using (COVES) as a cutoff value.

Based on Cochrane Handbook recommendations regarding publication bias assessment [11], at least 10 studies are needed to be included in the meta-analysis to assess publication bias. Therefore, we didn’t assess the publication bias.

## Results

### Search results

**Fig. 1:**
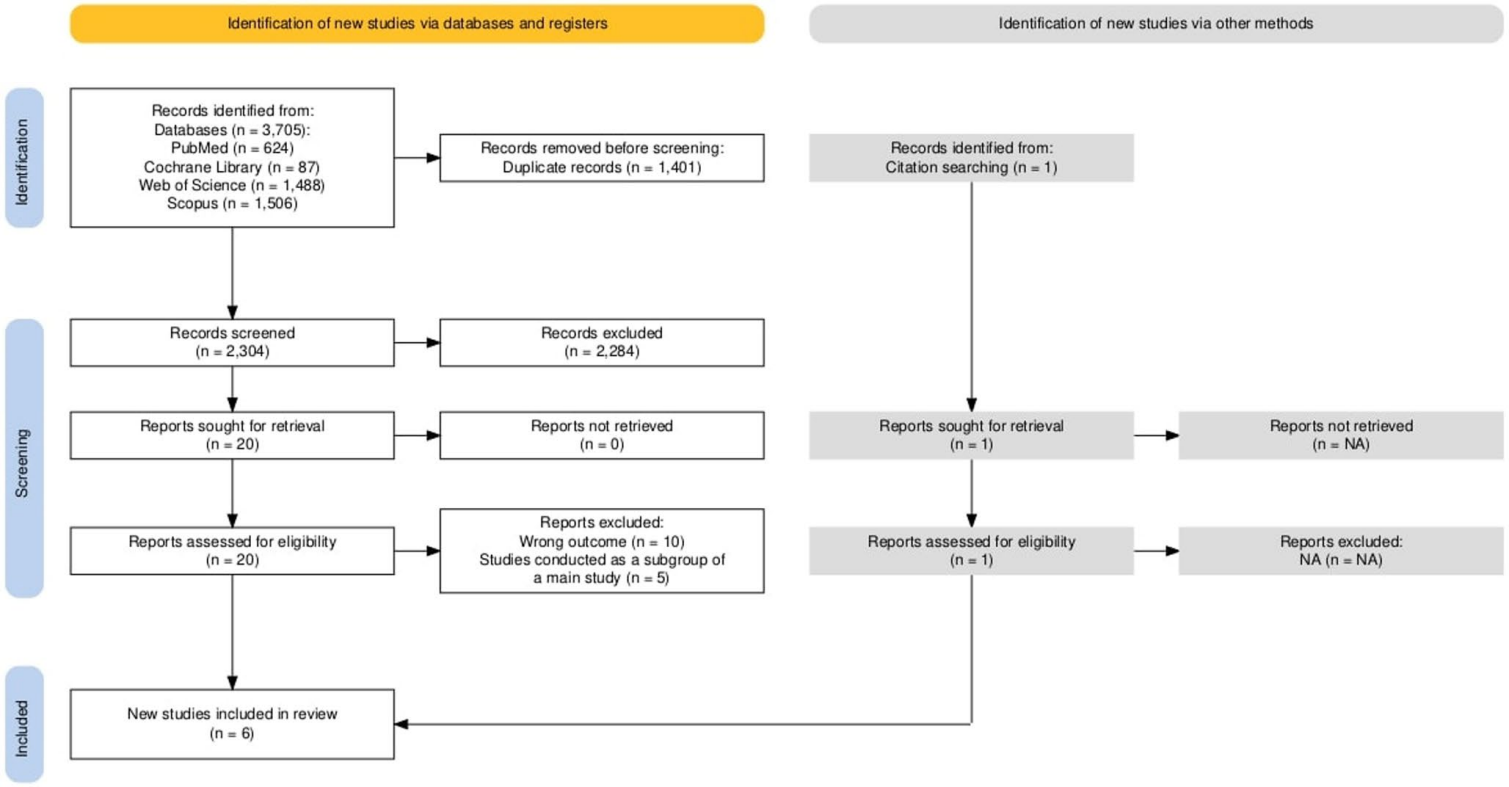
PRISMA flow chart of the screening process

A preliminary search through the predefined databases retrieved 3705 studies. After removing 1401 duplicates. 2304 records remained for title and abstract screening. Following this stage, 2284 records were excluded, leaving 20 studies for full-text review. Subsequent full-text screening led to the exclusion of 14 studies, ultimately, 6 studies met all inclusion criteria and were included in this review. The PRISMA flow diagram illustrates the search, selection, and exclusion processes and their corresponding reasons Fig. 1.

### Characteristics of included studies

A total of six studies were included in this meta-analysis [6, 8, 14–17]. These studies comprised a total of (2249) patients, with (1251) patients in the unfavorable VO group and (998) in the favorable VO group. The studies predominantly included retrospective cohort designs, with two post-hoc analyses of major trials (RESCUE-BT and ESCAPE NA-1) [18, 19]. Most studies defined favorable VO using the COVES score, with thresholds of > 2 [8, 14, 16, 17]. Meanwhile, Li et al. [6], and Gong et al. [15] identified favorable VO as COVES > 3. The included studies covered diverse populations across China, the USA, Germany, and other international sites. They highlighted the prognostic value of (VO) profiles in predicting clinical outcomes post-endovascular treatment (EVT). Favorable VO profiles were consistently associated with improved functional independence and reduced risk of complications such as intracerebral hemorrhage (ICH). Conversely, unfavorable VO was linked to higher rates of hemorrhagic transformation and worse clinical outcomes. However, we identified some studies have the same inclusion criteria originating from two comprehensive stroke centers (University Medical Center Hamburg-Eppendorf, Germany, and Stanford, University Hospital, California) Given the potential for these studies to include overlapping patient data [5, 7, 20], excluding these studies likely mitigated the risk of bias that could arise from analyzing non-independent data and prevent undue influence of a single patient group on the overall meta-analysis estimate. Ultimately, we selected only one representative study containing the most intended outcomes for inclusion in the quantitative analysis [16].

The characteristics of included studies are presented in (Tables 1 and 2).

**Table 1:** Characteristics of the studies included in the meta-analysis

Study ID	Country	Study period	Study design	Definition of favorable VO	EVT Time window	Conclusion
Chu et al. 2023	China	2018–2022	Retrospective Cohort	COVES > 2	within 24 h	Favorable peak venous VO profiles on mCTA might be a promising biomarker in predicting the good outcome in patients with AIS after EVT.
Gong et al. 2024	China	2019–2023	Post-hoc analysis of RESCUE-BT trial	COVES > 3	6–24 h	The robust VO profile indicated by COVES 4–6 could promote the frequency of functional independence among AIS-LVO patients receiving EVT in the late window, and the prognostic value of VO was independent of the arterial collateral status.
Jiang et al. 2024	China	2019–2023	Retrospective Cohort	COVES > 2	within 24 h	This study demonstrated the potential mediating effects of severe ICH for the beneficial effect of favorable VO on clinical prognosis among patients with AIS-LVO who underwent EVT
Li et al. 2023	China	2017–2022.	Retrospective Cohort	COVES > 3	within 24 h	Microvascular dysfunction is independently associated with unfavorable VO. The microvascular dysfunction represented by increased Ve may be an important mechanism of impaired venous collateral circulation.
Winkelmeier et al. 2022	USA& Germany	2013–2021	Retrospective Cohort	COVES > 2	within 16 h after symptom onset	Unfavorable VO was associated with the occurrence of hemorrhagic infarction and parenchymal hematoma, both related to worse clinical outcomes
Bala et al. 2022	The ESCAPE NA-1 trial was done at 48 acute care hospitals in Canada (14 sites), the USA (19), Germany (five), Australia (two), South Korea (four), Sweden (one), Ireland (two), and the UK (one	2017–2019	Post hoc analysis of the ESCAPE NA-1 trial	COVES > 2	Time from last seen well less than 12 h	Poor venous opacification on computed tomography angiography is strongly associated with an increased risk of PH and worse clinical outcomes after endovascular treatment, and therefore it may be used as a tool for risk stratification in patients with stroke

*VO *venous outflow, *COVES* cortical vein opacification Score, *EVT* endovascular treatment, *mCTA* multiphasic CT angiography, *AIS-LVO* acute ischemic stroke large vessel occlusion

**Table 2:** Baseline characteristics of patients

Study ID	Age^*^		Male^#^		Baseline NIHSS^*^		ASPECTS*		Occlusion site^#^		Good arterial collaterals^#^		(TICI 2b-3)^#^	
	VO+	VO-	VO+	VO-	VO+	VO-	VO+	VO-	VO+	VO-	VO+	VO-	VO+	VO-
Bala et al. 2022	70 (59–79)	71 (61–81)	132 (51)	139 (48,6)	15 (11–20)	18 (15–21)	8 (7–9)	8 (7–8)	ICA = 36 (13.9) MCA-M1 = 207(80.2) MCA-M2 = 15 (5.8)	ICA = 88 (30.8), MCA-M1 = 194 (67.8) MCA-M2 = 4 (1.4)	57 (22.3)	33 (11.7)	231 (89.5)	242 (84.9)
Gong et al. 2024	65 (55–71)	67 (56–75)	79 (64.8)	132 (62.9)	13 (10–18)	16 (11–19)	7 (7–9)	7 (6–9)	ICA = 16 (13.1) MCA-M1 = 8(65.6) MCA-M2 = 26 (21.3)	ICA = 35 (16.7) MCA-M1 = 146 (69.52) MCA-M2 = 29 (13.8)	NA	NA	110 (90.2)	189 (90.0)
Jiang et al. 2024	68 (58–76)	70 (61–78)	182 (60.3)	101 (47.4)	12 (8–18)	17 (12–20)	7 (6–9)	6 (4–8)	ICA = 90 (29.8) MCA-M1 = 162 (53.6) MCA-M2 = 50 (16.6)	ICA = 79 (37.1) MCA-M1 = 112 (52.6) MCA-M2 = 22 (10.3)	NA	NA	284 (94.0)	194 (91.1)
Li et al. 2023	66 (60–79)	68 (62–77)	23 (58.97)	40 (63.49)	10 (5–14)	12 (9–16)	9 (9–10)	8 (8–10)	ICA = 11 (28.20) MCA-M1 = 23 (58.97) MCA-M2 = 5 (12.82)	ICA = 16 (25.39) MCA-M1 = 41 (65.08) MCA-M2 = 6 (9.52)	25 (64.10)	13 (20.63)	34 (87.18)	52 (82.54)
Winkelmeier et al. 2022	70.1 (14.3)	74.3 (13.8)	140 (56.7%)	167 (43.7%)	11 (7–17)	17 (12–20)	NA	NA	NA	NA	203 (82.2%)	170 (44.5%)	216 (87.4%)	291 (76.2%)

*VO+* favorable venous outflow, *VO *unfavorable venous outflow, *NIHSS *National Institutes of Health Stroke Scale, *ASPECTS *Alberta Stroke Program Early CT Score, *ICA *internal carotid artery, *MCA* middle cerebral artery, *TICI *thrombolysis in cerebral infarction, *TICI 2b-3 *good recanalization, *NA *not applicable

(*); Data are presented as the median with interquartile [IQR] or (#); numbers of patients with percentages in parentheses (%)

### Risk of bias and quality assessment

**Fig. 2:**
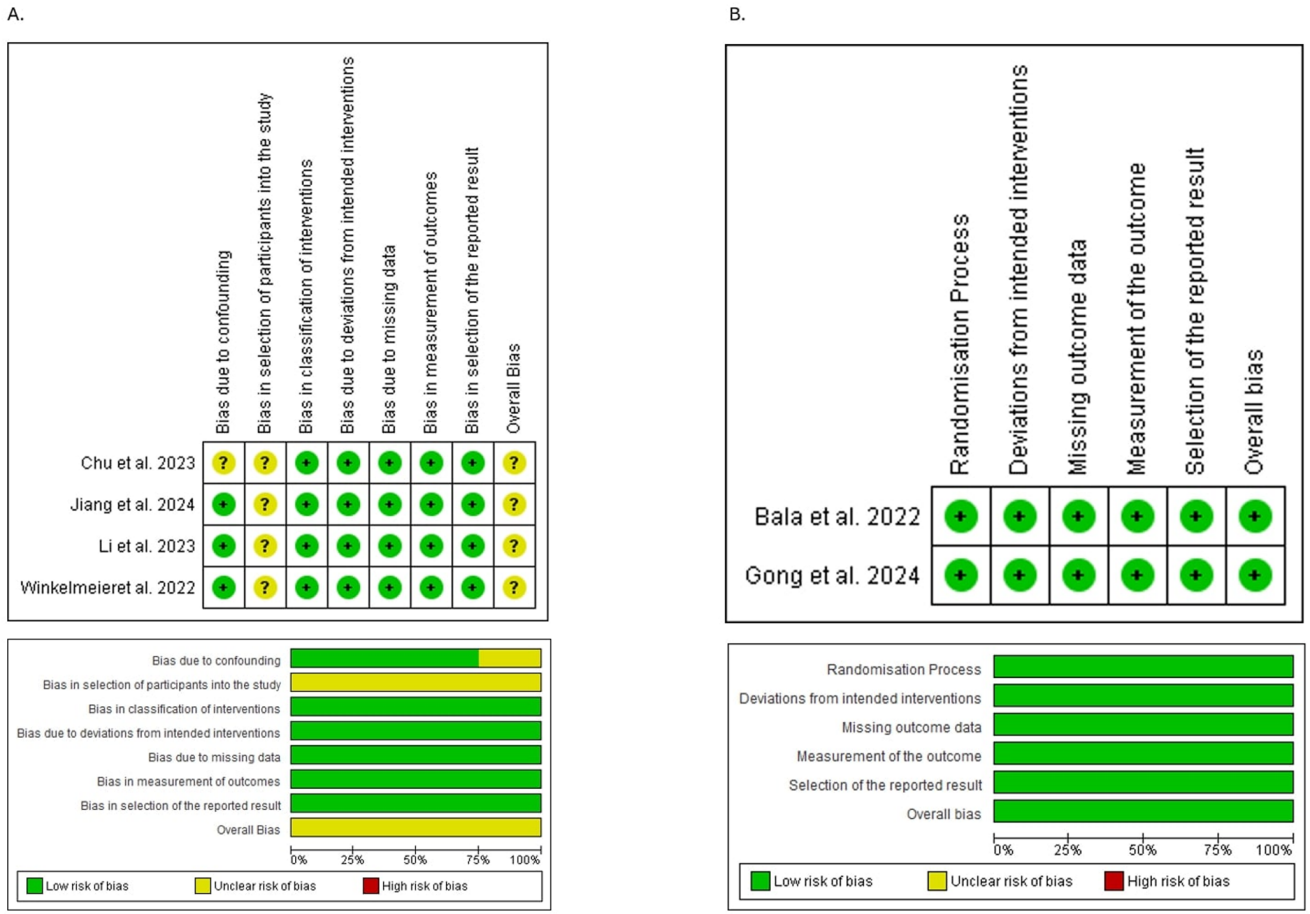
Quality assessment for the included studies. (**A**); ROBINS-I for observational studies, (**B**); ROB-2 for randomized clinical trials

Of the two included trials, all were judged to have a “low” risk of bias, as assessed by the RoB-2 tool. The four cohort studies were assessed using ROBINS-I, of which all were deemed to have “some concerns” of bias, primarily due to confounding in the selection of participants. Detailed quality assessment figures are presented in Fig. 2.

### Clinical outcomes

#### Functional independence (Modified Rankin scale at 90 days (mRS 0–2))

**Fig. 3:**
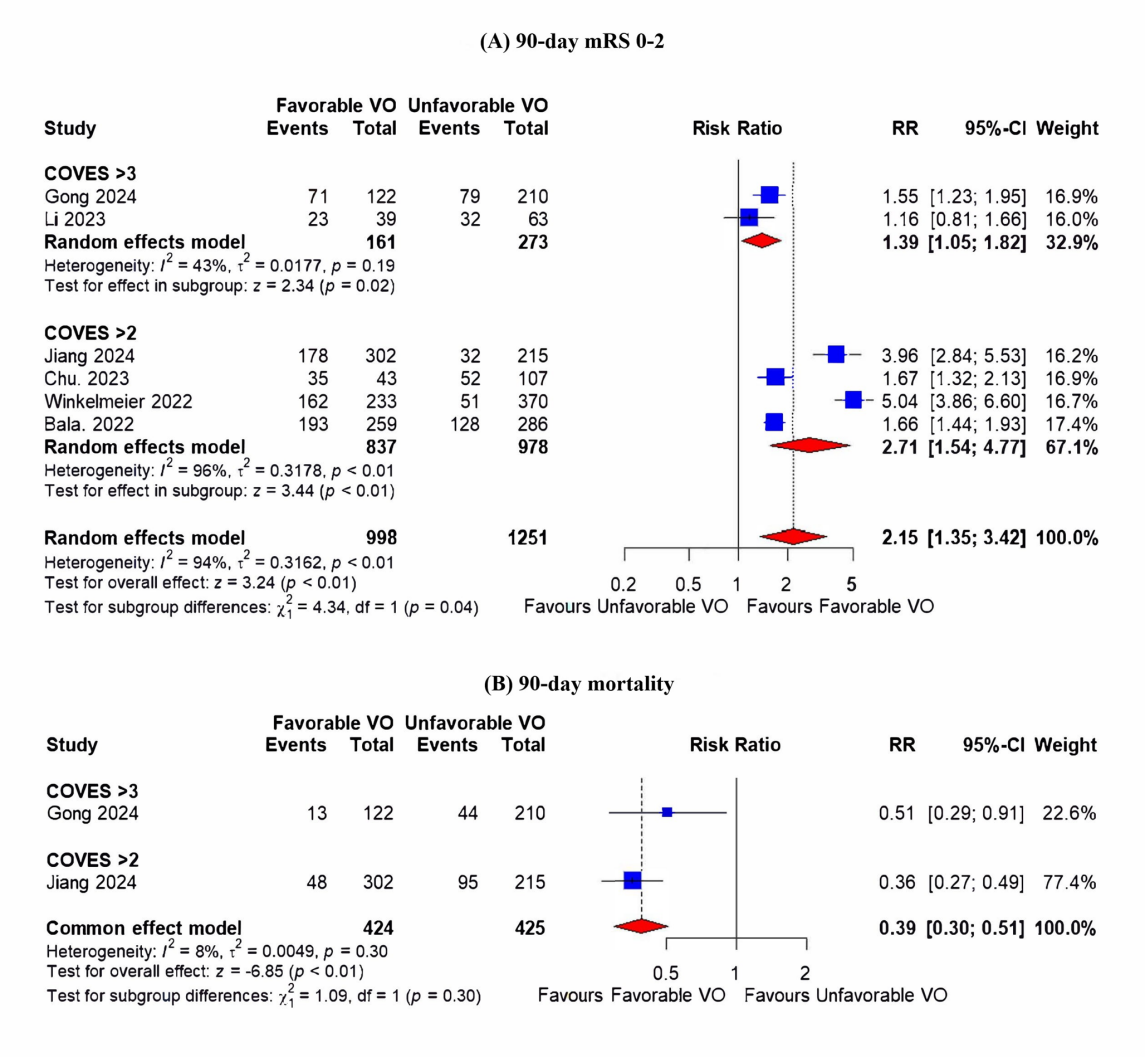
presents forest plots analyzing clinical outcomes after 90 days. These plots incorporate a sub-group analysis between studies that evaluated favorable venous outflow based on cortical venous opacification score (COVES). (**A**) functional independence (mRS 0–2); (**B**) 90-day mortality

The analysis included six studies. Favorable VO was associated with significantly higher rates of functional independence compared to unfavorable VO (RR = 2.15; 95% CI: 1.35, 3.42; *p* = 0.01) (Fig. 3A). However, the result was significantly heterogeneous (I^2^ = 94%, *p* < 0.01). Sensitivity analysis couldn’t resolve the high heterogeneity (Figure S1).

### 90-day mortality

The analysis included two studies. Favorable VO was associated with a significantly lower incidence of 90-day mortality (RR = 0.39; 95% CI: 0.30, 0.51; *p* < 0.01) (Fig. 3B). The result was homogenous (I^2^ = 8%, *p* = 0.30).

### Follow-up imaging

#### Parenchymal hematoma

**Fig. 4:**
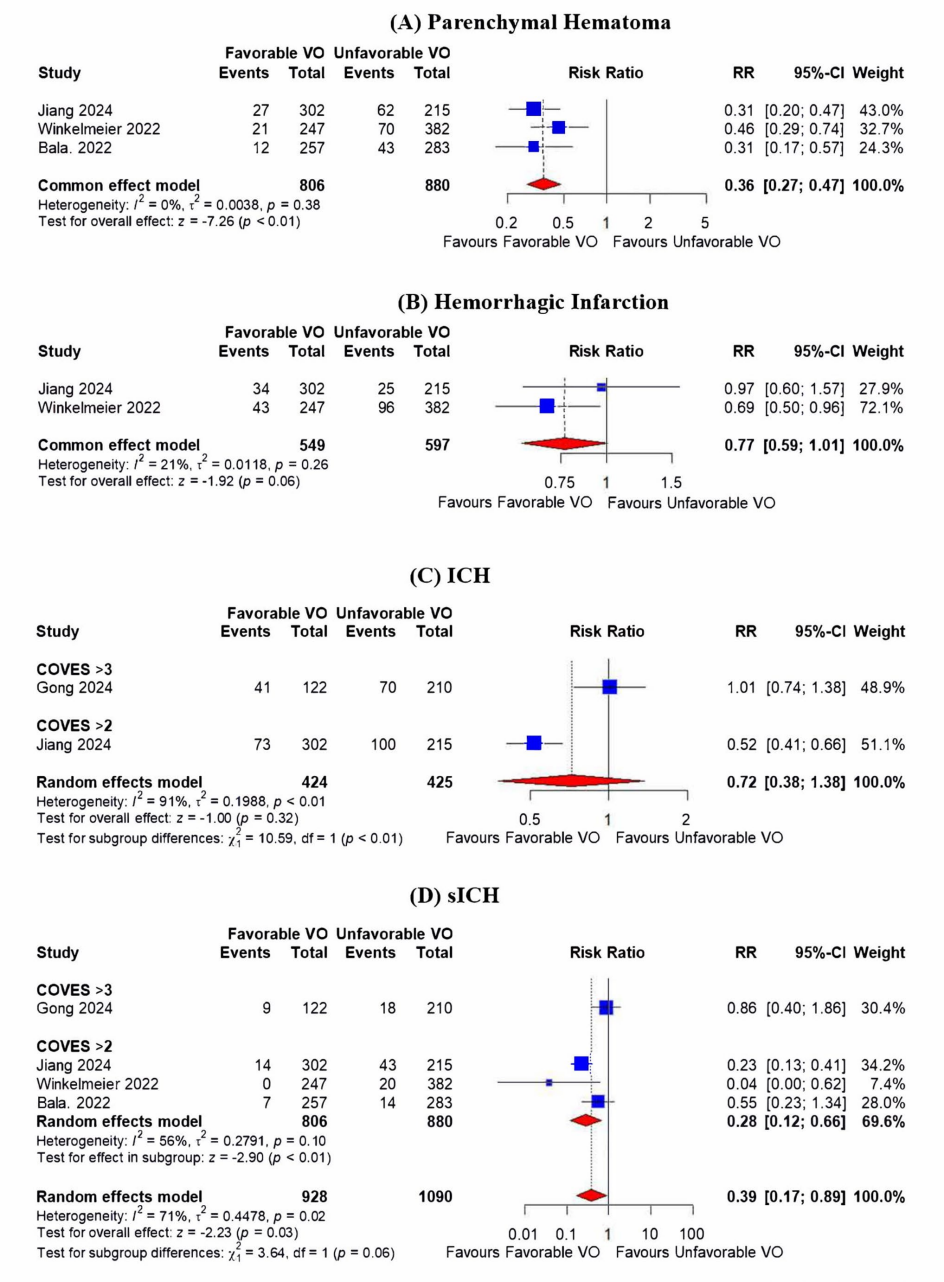
presents forest plots analyzing follow-up imaging following endovascular thrombectomy. (**A**) parenchymal hematoma, (**B**) hemorrhagic infarction, (**C**) any intracranial hemorrhage, (**D**) symptomatic intracranial hemorrhage (sICH)

The analysis included three studies. Favorable VO was associated with a significantly lower incidence of parenchymal hematoma compared to unfavorable VO (RR = 0.36; 95% CI: 0.27, 0.47; *p* < 0.01) (Fig. 4 A). The result was homogenous (I^2^ = 0%, *p* = 0.38).

#### Hemorrhagic infarction

The analysis included two studies. Favorable VO was associated with a lower incidence of hemorrhagic infarction; however, it was not significant (RR = 0.77; 95% CI: 0.59, 1.01; *p* = 0.06) (Fig. 4B). The result was homogenous (I^2^ = 21%, *p* = 0.26).

#### Any intracranial hemorrhage

The analysis included two studies. Favorable VO did not differ from unfavorable VO regarding any intracranial hemorrhage (RR = 0.72; 95% CI: 0.38, 1.38; *p* = 0.32) (Fig. 4C). However, the result was significantly heterogeneous (I^2^ = 91%, *p* < 0.01). Sensitivity analysis could not be done due to the small number of studies included in the analysis.

#### Symptomatic Intracranial Hemorrhage (sICH)

The analysis included four studies. Favorable VO was associated with a significantly lower incidence of hemorrhagic infarction (RR = 0.39; 95% CI: 0.17, 0.89; *p* = 0.03) (Fig. 4D). The result was heterogeneous (I^2^ = 70%, *p* = 0.02). Sensitivity analysis showed that the result was homogenous after excluding Gong et al. 2024 (I^2^ = 56%) and the pooled estimate was significant (RR = 0.28; 95% CI: 0.12, 0.66; *p* < 0.01) (Figure S2).

### Subgroup analysis

Subgroup analysis revealed that favorable VO was associated with less promising results regarding functional independence, intracranial hemorrhage, and sICH in studies that defined favorable VO as COVES > 3 compared to those that defined it as COVES > 2. The subgroup difference was significant (*p* < 0.05) for functional independence and any intracranial hemorrhage (Figs. 3A and 4C), but it was marginally insignificant (*p* = 0.06) in terms of sICH (Fig. 4D).

## Discussion

This meta-analysis demonstrated significantly better functional independence in stroke patients with favorable venous outflow. Additionally, a lower incidence of adverse events associated with thrombectomy, like hemorrhagic infarction, parenchymal hematoma, and intracranial hemorrhage, was noted in the favorable VO group. Lower 90-day mortality rates were also observed in the good VO group. Significantly better patient outcomes were noted when the threshold of favorable VO was COVES > 2 as compared to COVES > 3.

Favorable VO was found to be significantly associated with functional independence, defined as mRS scores (0–2). Our findings are similar to those noted in the study by Adusumilli et al., where greater “functional independence” was noted in those with good venous outflow profiles [3], and Faizy et al., in which favorable VO was associated with good functional outcomes (OR: 8.9, 95%CI: 5.3, 14.9; *P* < 0.001) [20]. A theory is that venous outflow acts as a surrogate marker for tissue perfusion; severe tissue damage results in necrosis and release of inflammatory markers. These contribute to higher leakage from blood vessels, resulting in edema, elevated interstitial pressure, and consequently poorer venous outflow [21]. Thus, favorable VO implies a high functional status of collaterals and perfusion at the site of ischemic stroke. Multiple studies have enforced the need for early initiation and adequate collateral circulation to aid in patient recovery [22]– [23]. Additionally, the recurrence of stroke is decreased significantly by the development of good cerebral collaterals in ischemic stroke [24]. It can be concluded based on our findings, that favorable VO before intervention can be a marker for functional independence following thrombectomy.

Finally, the 90-day mortality rates were found to be lower in those patients with favorable VO, thus implying the predictive role of VO in measuring immediate and long-term outcomes. Findings in this study are substantiated by Yedavalli et al., who noted higher mortality in those with slower venous transit, implying insufficiency of venous flow [25]. Furthermore, Ryu et al. noted an association between incomplete transverse sinus shape and high mortality rates following malignant middle cerebral artery infarction [26]. Favorable VO implies successful microvascular cerebral blood flow, thus preventing further ischemia-mediated neuronal loss, finally culminating in lesser mortality risks.

In addition to neurological improvement, favorable VO was significantly associated with a lower incidence of parenchymal hematoma and symptomatic intracranial hemorrhage. Across all included studies similar conclusions were drawn on the effectiveness of VO in predicting adverse events, with Jiang et al. quantifying a reduction of 8% in the incidence of parenchymal hematoma (95%CI: 0.9–19%) [8]. A lower incidence of hemorrhagic infarction was noted as well, albeit with insignificance. This could be due to fewer studies and the small patient population. Further, while Jiang et al. noted a significantly reduced incident ICH and sICH in the favorable VO group, these findings did not resonate with Gong et al. [15]. This could be due to the different basis of defining “favorable VO” in both studies; while Gong et al. considered favorable venous outflow in those with COVES score 4–6, Jiang et al. quantified favorable VO with COVES score 3–6. On subgroup analysis, a stronger association was found between COVES score > 2. and good functional outcomes and lower incidence of adverse events when compared to COVES > 3. COVES is an independent marker of collateral filling [4]. Lakhani et al. noted an association of COVES score with standard tests of grading collateral function when good venous outflow was assigned as COVES ≥ 3 [27]. Thus, the use of COVES (3–6) as the definition of favorable VO has better implications and associations than COVES (4–6).

### Implication for future research

While venous outflow (VO) has demonstrated prognostic utility in predicting functional outcomes and hemorrhagic complications after thrombectomy, several limitations must be acknowledged when considering VO as a standalone imaging biomarker. The cerebral venous system exhibits substantial anatomical variability, particularly in the configuration of cortical veins and dural sinuses. Variations such as asymmetry, hypoplasia, or aplasia of the transverse sinuses can impact VO assessment independently of actual perfusion or collateral status, potentially leading to misclassification [28]. Scoring methods like COVES rely on manual grading by experienced neuroradiologists, introducing inter- and intra-observer variability [29]. The lack of automated tools limits clinical applicability and reproducibility, particularly in acute stroke workflows where rapid decision-making is essential [30]. Additionally, Single-phase CTA captures only one time point and may fail to detect delayed or incomplete venous filling, underestimating VO, especially in patients with slow collateral flow. In contrast, multiphase CTA provides dynamic assessment across arterial to late venous phases, enhancing the detection of delayed drainage and improving VO evaluation [31]. However, the use of multiphase CTA is not yet widespread, and heterogeneity in acquisition protocols across centers can affect the comparability of VO measurements.

Given these challenges, VO should be interpreted alongside arterial collateral scores, perfusion imaging, and clinical context. Further research is needed to validate automated VO quantification tools, define standardized imaging protocols, and establish consensus thresholds to improve VO’s reliability as a biomarker in acute stroke care.

Multiphasic computed tomography angiography (CTA) should be employed to comprehensively assess venous outflow. Sub-studies are needed to characterize the prognostic significance of VO in predicting patient outcomes across different ischemic stroke subtypes. Furthermore, future trials should specifically investigate the predictive value of VO in patients undergoing endovascular treatment (EVT) within the late window (beyond 6 h from stroke onset).

### Limitations

The inclusion of only six studies limits the wide generalization of results. Additionally, the inclusion and pooling of observational studies and post-hoc analyses of randomized trials may have resulted in bias in our findings. Most of the included studies were retrospective in design, which increases the risk of selection bias and confounding. We conducted a rigorous quality assessment to control for bias, and all depicted “some concerns”, suggesting moderate biases. A majority of our included studies were conducted in China, therefore limiting the applicability of our findings to this subset of the population. Significant heterogeneity was observed in the analysis of functional independence (I² = 94%) and any intracranial hemorrhage (I² = 91%). This heterogeneity may stem from differences in study design, population characteristics, imaging protocols, and definitions of favorable VO. Although subgroup and sensitivity analyses were performed, heterogeneity could not be fully resolved, indicating variability in effect estimates.

Included studies utilized different thresholds of the COVES score to define favorable VO. While most studies defined favorable VO as COVES > 2, others used > 3. This inconsistency may influence the pooled results and limit comparability across studies. The subgroup analysis revealed that the prognostic value of favorable VO was more pronounced when defined as COVES > 2, suggesting that the definition used can significantly impact outcome interpretation and we couldn’t perform dose response analysis due to the presence of only two categories. Some clinical outcomes, such as 90-day mortality, hemorrhagic infarction, and any intracranial hemorrhage, were evaluated in only two studies, limiting the statistical power and generalizability of those findings. Furthermore, sensitivity analyses could not be performed in these cases, precluding further exploration of heterogeneity or robustness of the effect estimates. Differences in imaging modalities, timing of imaging, and interpretation of COVES scores could introduce measurement bias. Standardized protocols for assessing VO across institutions were not reported, possibly contributing to inter-study variability.

## Conclusion

Favorable venous outflow (VO) profiles are associated with good neurological outcomes, a reduced incidence of parenchymal hematoma, lower mortality rates, and decreased symptomatic intracranial hemorrhage (sICH) following reperfusion therapy. These findings suggest that VO profiles may serve as robust prognostic imaging biomarkers to predict patient outcomes.

## Electronic supplementary material

Below is the link to the electronic supplementary material.

Supplementary Material 1

## Data Availability

No datasets were generated or analysed during the current study.
