# Supplementary material for: Prognostic value of cerebral venous outflow profiles for outcomes prediction following reperfusion therapy in acute ischemic stroke: a meta-analysis
Source: Neuroradiology. 2025 Jul 8;67(9):2283–94. doi: 10.1007/s00234-025-03688-w (PMC12546523; doi:10.1007/s00234-025-03688-w)

Supplementary Table S1: Search strategy across all included databases

| Database | Search Terms | Search Field | Search Results |
| --- | --- | --- | --- |
| PubMed | (thrombectomy* OR embolectomy* OR “endovascular thrombectomy” OR “mechanical thrombectomy” OR endovascular OR “Vessel Reperfusion” OR thrombolysis) AND (stroke OR “acute ischemic stroke” OR “hemorrhagic stroke” OR “transient ischemic attack” OR “cerebrovascular accident” OR CVA OR “apoplexy” OR “cerebral accident” OR “cerebral infarction” OR “brain attack” OR “Cortical Vein Opacification Score” OR COVES) AND (“venous outflow” OR venous^*^ OR “poor venous outflow” OR “favorable venous outflow”) | All Fields | 624 |
| Cochrane | (thrombectomy* OR embolectomy* OR “endovascular thrombectomy” OR “mechanical thrombectomy” OR endovascular OR “Vessel Reperfusion” OR thrombolysis) AND (stroke OR “acute ischemic stroke” OR “hemorrhagic stroke” OR “transient ischemic attack” OR “cerebrovascular accident” OR CVA OR “apoplexy” OR “cerebral accident” OR “cerebral infarction” OR “brain attack” OR “Cortical Vein Opacification Score” OR COVES) AND (“venous outflow” OR venous^*^ OR “poor venous outflow” OR “favorable venous outflow”) | All Fields | 87 |
| Web of Science | (thrombectomy* OR embolectomy* OR “endovascular thrombectomy” OR “mechanical thrombectomy” OR endovascular OR “Vessel Reperfusion” OR thrombolysis) AND (stroke OR “acute ischemic stroke” OR “hemorrhagic stroke” OR “transient ischemic attack” OR “cerebrovascular accident” OR CVA OR “apoplexy” OR “cerebral accident” OR “cerebral infarction” OR “brain attack” OR “Cortical Vein Opacification Score” OR COVES) AND (“venous outflow” OR venous^*^ OR “poor venous outflow” OR “favorable venous outflow”) | All Fields | 1448 |
| SCOPUS | (thrombectomy* OR embolectomy* OR “endovascular thrombectomy” OR “mechanical thrombectomy” OR endovascular OR “Vessel Reperfusion” OR thrombolysis) AND (stroke OR “acute ischemic stroke” OR “hemorrhagic stroke” OR “transient ischemic attack” OR “cerebrovascular accident” OR CVA OR “apoplexy” OR “cerebral accident” OR “cerebral infarction” OR “brain attack” OR “Cortical Vein Opacification Score” OR COVES) AND (“venous outflow” OR venous^*^ OR “poor venous outflow” OR “favorable venous outflow”) | Title, Abstract, Keywords | 1506 |
| Date of Search | | | October 23, 2024 |

Supplementary Figure S1: Sensitivity analysis of functional independence


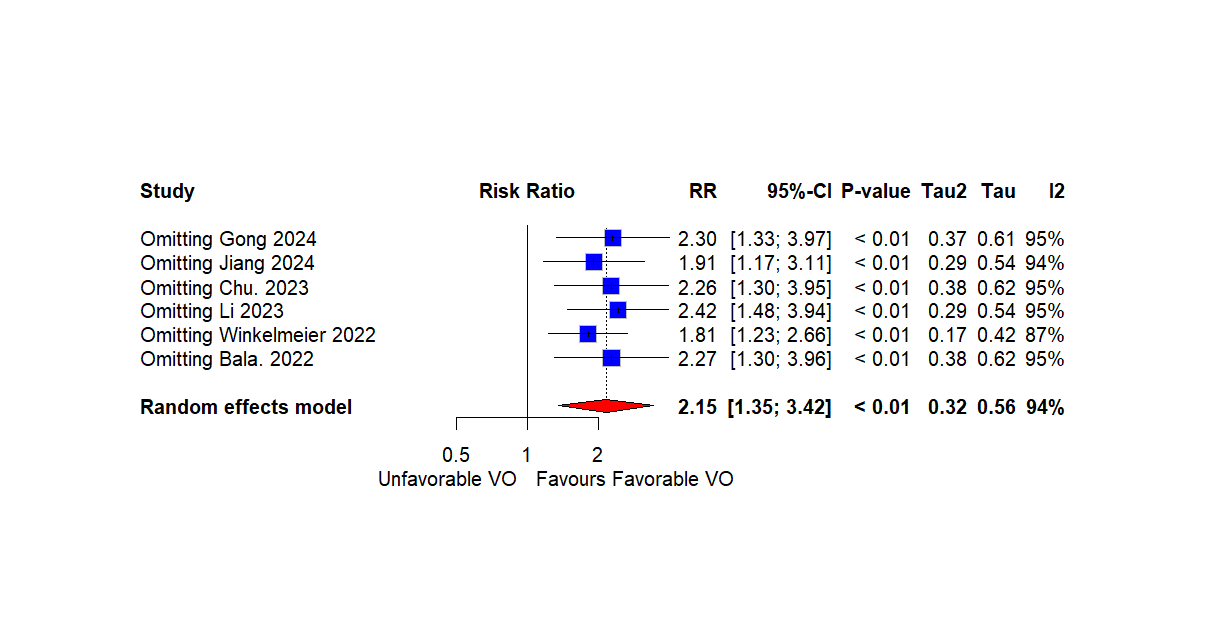


Supplementary Figure S2: Sensitivity analysis of event of symptomatic intracranial hemorrhage


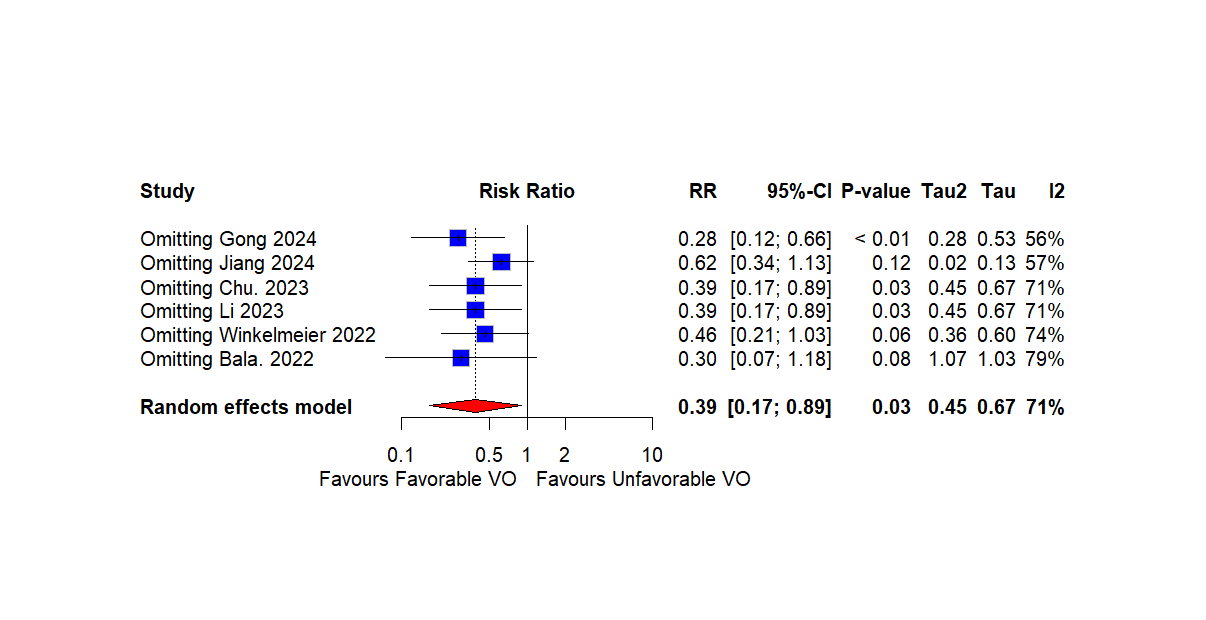

Supplement: Supplementary file 1 — Supplementary Material 1 [file 234_2025_3688_MOESM1_ESM.docx]
